# Maternal depressive symptom trajectories and associations with child feeding

**DOI:** 10.1186/s12889-024-19110-8

**Published:** 2024-06-19

**Authors:** Katelyn Fox, Chantelle N. Hart, Suzanne Phelan, Alison K. Ventura, Rena Wing, Elissa Jelalian

**Affiliations:** 1 Weight Control and Diabetes Research Center, The Miriam Hospital, 196 Richmond Street, Providence, RI 02903 USA; 2 Department of Psychology and Human Behavior, Alpert Medical School Brown University, 222 Richmond St, Providence, RI 02903 USA; 3 Department of Social and Behavioral Sciences & Center for Obesity Research and Education, College of Public Health Temple University, 3223 North Broad Street, Suite 175, Philadelphia, PA 19140 USA; 4 Department of Kinesiology and Public Health & Center for Health Research Bailey College of Science and Mathematics, California Polytechnic State University, 1 Grand Avenue, San Luis Obispo, CA 93407 USA

**Keywords:** Depressive symptoms, Feeding behaviors, Maternal responsiveness, Satiation cues

## Abstract

**Background:**

Responsive feeding, when caregivers attend to children’s signals of hunger and satiation and respond in an emotionally supportive and developmentally appropriate way, is associated with the development of healthy eating behaviors, improved diet quality, and healthy weight status for children. However, gaps in the literature remain on how factors, such as maternal depressive symptoms and child temperament, influence feeding interactions.

**Methods:**

This longitudinal secondary data analysis explored the association between maternal depressive symptom trajectory and child temperament with maternal feeding practices in women with obesity who participated in a prenatal lifestyle intervention trial. Mothers self-reported depressive symptoms at baseline, 35 weeks gestation, and 6, 12, and 18 months postpartum. At 18- and 24-months postpartum, mothers completed self-reported assessments of feeding practices and child temperament and completed in-home video-recorded meals with their child, coded using the Responsiveness to Child Feeding Cues Scale. We used group-based trajectory modeling to identify distinct trajectories of depressive symptoms and generalized regressions to assess the association between symptom trajectory group and feeding. We also explored interactions between depressive symptoms and child temperament.

**Results:**

Three distinct trajectories of depressive symptoms were identified: No-Minimal and Decreasing, Mild-Moderate and Stable, and Moderate-Severe and Stable. At 18-months, when compared to the No-Minimal and Decreasing group, membership in the Moderate-Severe and Stable group was associated with higher observed responsiveness to child satiation cues ($B$ =2.3, *95%CI =* 0.2, 4.4) and lower self-reported pressure to eat ($B$=-0.4, *95%CI*= -0.7, 0.0). When compared to the No-Minimal and Decreasing group, membership in the Mild-Moderate and Stable group was associated with higher self-reported restriction ($B$ =0.4, *95%CI* = 0.0,0.7). The associations between trajectory group membership and feeding practices did not reach statistical significance at 24 months. Associations between depressive symptoms and restriction were moderated by child effortful control at 18 months $(B=0.2, 95\% CI (0.0, 04)$) and surgency at 24 months $B=-0.3, 95\% CI (-0.6, 0.0)$).

**Conclusion:**

A Moderate-Severe and Stable depressive symptom trajectory was associated with more responsive feeding practices and a Mild-Moderate and Stable trajectory was associated with higher restrictive feeding. Preliminary evidence suggests that depressive symptoms impact mothers’ ability to match their use of restriction to the temperamental needs of their child.

**Supplementary Information:**

The online version contains supplementary material available at 10.1186/s12889-024-19110-8.

## Background

Fostering healthy eating behaviors early in childhood is a public health priority as food preferences and eating behaviors are established early in life and track into adulthood, influencing the development of obesity and diet-related diseases [1–3]. As children grow, their eating behaviors are shaped by the environment in which caregivers socialize their children around food [4–11]. Caregivers influence the feeding environment through their own emotions and behaviors, which are considered to fall along two dimensions, how responsive they are to their child and how demanding/directive they are when guiding their child’s behavior [12]. A feeding environment in which caregivers encourage child autonomy in eating while providing appropriate structure around food and mealtimes is associated with the development of healthy eating behaviors [13]. For example, caregivers can help children self-regulate eating in response to physiological feelings of hunger and satiation by attuning to children’s behavioral cues indicative of hunger and satiation [14–17]. Conversely, a feeding environment in which either the caregiver or child exerts excessive control may impair the development of self-regulation. For example, if caregivers attempt to control children’s eating behaviors by pressuring their children to eat or overtly restricting foods, their children may be more likely to develop problematic eating behaviors, have poor diet quality, and have higher weight status [16, 18–21]. Similarly, if a child has more control than is appropriate given their development and unique temperament, they may be more likely to consume a low-quality diet, consume larger portion sizes, and have higher weight status [22–25]. The establishment of a mutually responsive feeding relationship in which caregivers recognize their child’s cues and respond in an emotionally supportive manner that is contingent on the child’s cues/behavior, developmentally appropriate, and predictable is critical in supporting the development of healthy eating behaviors [1]. However, gaps in the literature remain on which factors influence caregivers’ different feeding behaviors.

One caregiver-level factor suggested to influence the use of supportive feeding behaviors and healthy child weight status is maternal depression [14, 26, 27]. Symptoms of depression, such as loss of interest or pleasure, low energy, and impaired cognitive ability can impact a mother’s ability to recognize, interpret, and appropriately respond to cues from their child during feeding. For example, mothers with depressive symptoms may be unable to recognize their child’s satiation cues which can be subtle (e.g., gaze aversion) or misinterpreted as unrelated to the feeding occasion (e.g., playing with food or increased interest in surroundings) [28]. Mothers may also struggle to engage with their child at meals to guide their eating behaviors, leading them to rely on strategies that require fewer cognitive and emotional resources such as taking control through pressure or restriction or disengaging from the feeding occasion entirely [29]. Additionally, depressive symptoms could impact a mothers’ ability to adjust the feeding environment or parenting strategies contingent on the individual and developmental needs of the child [14]. Several studies have provided support for these hypotheses, finding that maternal depressive symptoms are associated with decreased awareness of hunger and satiation cues and greater use of pressure to eat and restriction [30–32]. However, the evidence for this relationship is mixed; most studies have been cross-sectional and have relied solely on self-report of feeding behaviors [27, 33, 34].

One explanation for inconsistent associations is that the nature of maternal depression matters. For example, Marshall et al. found in a sample of Latino farmworker families that both mothers who had chronic depressive symptoms and those with episodic but severe depressive symptoms exhibited less responsive feeding practices, and their children were more likely to develop obesity at a two-year follow-up [35]. This is concerning because less supportive feeding during this critical developmental window could have a lasting impact on children’s eating behaviors. Understanding the nature of maternal depression over time and how it impacts early child feeding is particularly important in women with obesity, who are at increased risk of experiencing peri and postpartum depression, and who are more likely to have a child that develops obesity [36, 37].

Therefore, the objective of this analysis was to identify depressive symptom trajectories of women with obesity who participated in a prenatal lifestyle intervention trial to prevent excessive weight gain during pregnancy and assess the association between trajectories and feeding. We hypothesized that there would be heterogeneity in the trajectories of depressive symptoms across mothers in this study and that trajectories that were persistently high or increasing in depressive symptoms would be associated with less responsive observed and self-reported feeding behaviors.

## Methods

### Sample

This was a secondary analysis of data from the Healthy Beginnings/ Comienzos Saludables (HB/CS) study [38, 39]. HB/CS was a controlled trial in which pregnant women were randomized to either a multicomponent intervention to reduce excess weight gain in pregnancy or a control group that received usual prenatal care. The study was conducted at two sites, California Polytechnic State University in San Luis Obispo, CA and The Miriam Hospital in Providence, RI. The HB/CS prenatal lifestyle intervention ended during pregnancy and resulted in a significant reduction in gestational weight gain but had no significant effect on mother or child weight from birth through 12 months [38]. HB/CS completed data collection in May 2016.

Mothers who participated in and completed the HB/CS study (*n* = 264) were invited to participate in Mealtime for Toddlers (MTT), an observational cohort assessing feeding interactions and child weight status [39]. MTT exclusion criteria for child participants were chronic health conditions that could affect feeding or growth, significant food allergies or dietary restrictions that could significantly affect food provided and/or the feeding interaction, a diagnosed psychiatric condition or significant developmental delay that could affect the feeding interaction or participation in the study. Interested mothers attended an orientation in which study procedures were described and written consent was obtained (*n* = 163). Families completed one-week assessments when the child was 18 months and 24 months old during which participating mothers completed study questionnaires, dyads were asked to have a dinner or evening meal video recorded in their homes, and dyads were also weighed and measured for length/height. A final assessment was completed at 36 months during which only height and weight were obtained. Enrollment was rolling. All procedures were approved by the Institutional Review Boards at the Miriam Hospital and California Polytechnic State University in accordance with the Declaration of Helsinki. The MTT study was also conducted in collaboration with Temple University whose Institutional Review Board determined it to be exempt due to no participant enrollment or data collection being done at this site and that data transferred and analyzed at Temple was considered by the IRB to be de-identified.

### Measures

Depressive Symptoms: Mothers completed the Beck Depression Inventory II (BDI-II) to assess the presence and severity of depressive symptoms in the past 2 weeks at HB/CS baseline (9–15 weeks gestation), 35 weeks’ gestation, and after delivery at 6, 12, 18, and 24 months [40]. This 21-item measure is widely used and has been validated in diverse populations [41]. Higher total scores indicate higher severity of depressive symptoms. Scores from 0 to 9 suggest no or minimal depression; scores from 10 to 18 indicate mild to moderate depression; scores from 19 to 29 indicate moderate to severe depression; and scores from 30 to 63 indicate severe depression [42]. The measure had good internal reliability in this sample (Cronbach’s alpha = 0.9).

Responsiveness to child satiation cues was assessed in the video recorded meals at the 18 and 24-month assessments. Trained research assistants coded maternal responsiveness to satiation cues using the Responsiveness to Child Feeding Cue Scale (RCFCS) and rated mothers on a 5-point Likert scale (1 highly unresponsive- highly responsive) [43]. Mothers were rated as more versus less responsive based on the response latency to both the number and intensity of toddler-displayed satiation cues. Mothers who respond to earlier and more subtle satiation cues (e.g., slows or pauses pace of feeding) are rated as having higher responsiveness than those who respond to later and more overt child satiation cues (e.g., pushing plate away, playing with food).

20% of videos were coded by two or more trained coders who were fluent in the language spoken during the meal (English or Spanish) to determine interrater reliability. Three raters coded the videos recorded in English and two coded the videos recorded in Spanish. Intraclass correlation coefficients (ICCs) were used to determine reliability for maternal responsiveness ratings, which was adequate (ICC = 0.58 for videos in English and 0.60 for videos in Spanish).

Mothers also completed the Child Feeding Questionnaire (CFQ) at the 18 and 24-month assessments [44]. This 31-item survey assesses reported attitudes, beliefs, and practices relating to child feeding. Items were rated on a 5-point Likert scale with higher scores indicating greater endorsement of the behavior. For this analysis, we assessed the two subscales related to attitudes and practices regarding the use of coercive/controlling feeding strategies; pressure to eat and restriction. The pressure to eat subscale reflects a tendency to pressure their child to eat more food, “My child should always eat all the food on their plate”. The restriction subscale reflects the extent to which parents restrict access to foods, “I intentionally keep foods out of my child’s reach”. The CFQ also assesses caregivers’ concern for their child’s risk of overweight which was included as a covariate as it has demonstrated a consistent association with caregivers’ use of restriction. We calculated scores by taking the mean of each item in a subscale. The CFQ had acceptable-good internal reliability in this sample (Cronbach α = 0.7–0.9).

Child Characteristics: Mothers completed the Infant Behavior Questionnaire-Revised Very Short Form (IBQR VSF) which measures three dimensions of temperament: effortful control, surgency/extraversion, and negative affectivity [45]. This 37-item measure has established validity among diverse groups [43]. The effortful control subscale assesses the ability of a child to control attention and behavior, the surgency subscale measures the degree to which a child approaches novel stimuli, expresses positive emotions, is physically/vocally active, and is sensitive to stimuli in the environment. The negative affectivity subscale measures the degree to which a child expresses sadness, fear, and distress. Mothers rated how often their child engaged in behaviors for each subscale (e.g., Play with one toy for 5 to 10 min, move quickly toward new objects, show distress when tired) on a scale from 1 (Never) to 7 (Always) and a mean score was calculated for each subscale.

Trained research staff collected child anthropometric measurements using standardized methods [46]. Weight was obtained in light clothing without shoes. Recumbent length was measured at the 18-month assessment and standing height was measured at the 24-month assessment. Body Mass Index (BMI) z-scores were calculated based on the child’s age and sex using the World Health Organization growth standards [47].

Sociodemographic Characteristics: Mothers reported sociodemographic characteristics including their age, ethnicity, education level, household income, as well as child’s age and gender. Participants self-identified ethnicity as Hispanic/Latino or not Hispanic Latino and race as American Indian or Alaska Native, Asian, Black, Native Hawaiian or Pacific Islander, White, or Other. Mothers also completed the 2-item household food insecurity screener [48].

### Analysis

We conducted group-based trajectory modeling (GBTM) using the R lcmm package (Proust-Lima, 2017) to model depressive symptom trajectory patterns in MTT participants with more than one postpartum BDI measurement (*n* = 151, 93% of full sample), using maximum likelihood estimation to account for missing data [49, 50]. We first normalized BDI scores to account for the left-skewed distribution commonly seen in the assessment of depressive symptoms. We then used a linear mixed model to identify the shape of the relationship between depressive symptoms over time. Based on previous literature we anticipated significant individual variability, with 2–4 distinct trajectories of depressive symptoms [51]. We then compared models predicting 1–5 clusters as a quadratic function of time, allowing growth to vary between classes. We assessed fit based on the following criteria: Minimize Bayesian Information Criterion (BIC), average posterior probability of assignment (APPA) ≥ 0.7 and nearer to 1, relative entropy nearer to 1, % smallest class ≥ 5, and visual evaluation of actual and predicted trajectories [52].

We then restricted the sample to MTT participants who had completed at least one feeding assessment (*n* = 111, 68% of the full sample). Of these participants, 29% were missing at least one key variable (feeding variable or covariate) at the 18-month assessment and 18% of participants were missing at least one key variable at the 24-month assessment. We assessed the correlation between missingness and study variables, sociodemographic characteristics, and study site (RI or CA) and found that missingness at the 18-month assessment was correlated with Black race (*r* = 0.2, *p* = 0.03), the RI site (*r* = 0.4, *p* < 0.0001), and effortful control (*r* = 0.2, *p* = 0.02) while missingness at the 24-month assessment was correlated with negative affectivity (*r*=-0.2, *p* = 0.02). Therefore, a complete case analysis would result in a sample with fewer Black mothers, and children with higher effortful control and lower negative affectivity. Variables were considered missing at random, at least partially explained by the limited initial funding and enrollment window of the study leading to some dyads not enrolling until after the 18-month visit. We imputed missing variables using a fully conditional specification algorithm with logistic regression for binary and ordinal variables (food insecurity, maternal responsiveness) and predictive mean matching for continuous variables (temperament, pressure to eat, restriction) including all study variables and maternal race as an auxiliary variable. We created 50 imputations, and the results of all subsequent analyses were pooled across imputations.

We compared sample characteristics across depressive symptom trajectory groups using generalized linear models for continuous variables and Chi-square and Fisher’s exact tests for categorical variables. We also explored changes in the time-varying variables (feeding, food insecurity, child weight, and temperament) using linear mixed models.

To assess the association between the depressive symptom trajectory group and feeding, we used generalized regressions assuming a normal distribution for continuous variables (pressure and restriction) and a multinomial distribution with a cumulative logit link for ordinal variables (maternal responsiveness). We identified covariates associated with food parenting in the literature a-priori including maternal age, ethnicity, education level, household income, and food insecurity, and concern for child weight status as well as the child’s age, gender, and BMI z-score [53–56]. We also adjusted for the intervention group (multicomponent weight management or usual care) and site (CA or RI). Child temperament variables were tested individually as candidate covariates in each model; however, inclusion did not change the main effect by greater than 10% and therefore temperament was not adjusted for in the final model.

### Post-hoc exploratory analysis

We also conducted an exploratory analysis to explore potential bidirectional associations between mothers and their children. We calculated Pearson correlation coefficients and used Fisher z-transformation to calculate the standard error so that estimates could be pooled across imputations. Because we were interested in the association between depressive symptoms and mothers’ responsiveness to their children, we then examined interaction effects between depressive symptoms and child temperament on the association with feeding variables. Due to the modest sample size and unequal groups identified using GBTM, we conducted this analysis using continuous mean BDI-II scores to maximize our power to detect moderation effects. We conducted a criterion power analysis using G-Plus and determined that our sample size was sufficient to detect a moderation effect (increase in variance explained by the model including the interaction term) with an alpha set at 0.1. We then conducted a simple slopes analysis to examine associations with an observed interaction effect.

## Results

### Depressive Symptom Trajectory groups

**Fig. 1:**
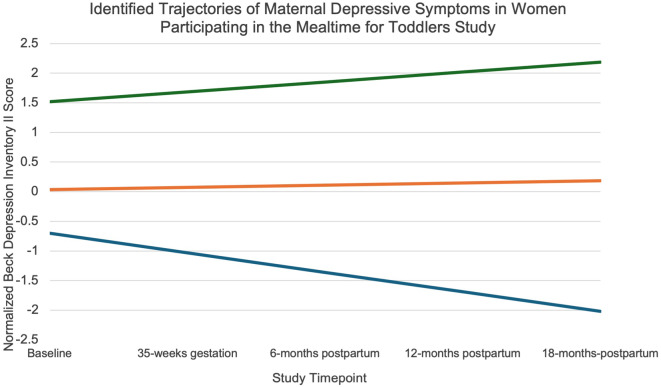
Group membership modeled using group-based trajectory modeling. 3 Group 1 represented in blue (*n* = 76, 48%), Group 2 represented in red (*n* = 56, 39%) Group 3 represented in green (*n* = 19,13%)

Linear mixed models revealed a significant fixed effect ($B$ =-0.12, *p-value* = 0.002) and random effect ($B$ =0.04, *p-value* = 0.049) of time on depressive symptoms. Of the 1–5 group models tested, the 3-group model best fit the data based on model fit (lowest BIC, satisfactory average posterior probability assignment, entropy, smallest group %, Table 1) and clinical significance (distinct mean depressive symptoms and % experiencing > minimal depressive symptoms (BDI-II score > 9) at each timepoint, Table 2). The first trajectory was characterized by no-minimal depressive symptoms at baseline that decreased significantly over time, the second trajectory was characterized by mild-moderate depressive symptoms at baseline that were stable over time, and the third trajectory was characterized by moderate-severe depressive symptoms at baseline that increased over time, however, the increase was not significant. The predicted probability of group membership was 48% No-Minimal and Decreasing, 39% Mild-Moderate and Stable, and 13% Moderate-Severe and Stable (Fig. 1).

**Table 1:** Model fit statistics comparing 1–5 class group-based trajectory models

Model	BIC	APPA		Entropy	% of sample in the smallest group
GBTM 1	2305.432		1	1	
GBTM 2	2154.311		0.9361401	0.8021281	35%
GBTM 3	2120.882		0.8859469	0.7530299	13%
GBTM 4	2124.448		0.8465606	0.7095939	12%
GBTM 5	2138.300		0.7335402	0.5944970	11%

Group based trajectory model (GBTM); Bayesian Information Criteria (BIC), Average Posterior Probability of Assignment (APPA)

**Table 2:** BDI-II1 scores over study period by depressive symptom trajectory groups

	Depressive Symptom Trajectory Groups^2^					
	No-Minimal and Decreasing		Mild-Moderate and Stable		Moderate-Severe and Stable	
	BDI II Mean ± SD	> minimal depressive symptoms^3^%	BDI II Mean ± SD	> minimal depressive symptoms^3^%	BDI II^3^ Mean ± SD	> minimal depressive symptoms^3^%
Baseline (*n* = 151)	5.5 ± 3.2	0	8.6 ± 5.0	12	17.8 ± 7.0	68
35 weeks gestation (*n* = 141)	6.0 ± 3.9	3	9.7 ± 4.3	17	17.2 ± 4.8	79
6 months post-partum (*n* = 145)	3.1 ± 3.0	1	9.7-±5.2	16	20.9 ± 7.3	88
12 months post-partum (*n* = 137)	2.6 ± 2.5	0	9.7 ± 5.0	21	19.9 ± 6.8	87
18 months post-partum (*n* = 53)	2.6 ± 2.3	0	10.4 ± 3.4	16	23.5 ± 11.7	67

*Note* Scores from 0 through 9 suggest no or minimal depression; scores from 10 through 18 indicate mild to moderate depression; scores from 19 through 29 indicate moderate to severe depression; and scores from 30 through 63 indicate severe depression [42]

^1^Beck Depressive Inventory II (BDI II) ^2^Group membership modeled using group-based trajectory modeling. ^3^ Greater than minimal depressive symptoms are defined as scores > 9 on the BDI II

### Sample characteristics

Table 3 presents the characteristics of the sample, which consisted of 111 ethnically and socioeconomically diverse mother/child dyads. Compared with the No-Minimal and Decreasing group, the Moderate-Severe and Stable group was younger (*p-value* = 0.02), less likely to be married (*p* = 0.04), and more likely to be experiencing food insecurity at 18-months (*p-value* = 0.01) and 24-months (*p-value* = 0.02). Compared to children of women in the No-Minimal and Decreasing group, children of women in the Moderate-Severe and Stable group had higher surgency at the 24-month assessment (*p-value* = 0.02). There were no other significant differences observed between groups.

### Changes in study variables over time

Self-reported and observed feeding variables at the 18 and 24-month timepoints are presented in Table 4. On average, child effortful control increased from the 18-month to 24-month assessment ($B$=0.3, *p-value* = 0.0006), with no time-by-trajectory group interaction. On average, child surgency was stable across time points ($B$= 0.0, *p-value* = 0.6), however, there was a significant time-by-trajectory group interaction, with the Moderate-Severe and Stable group associated with an increase in reported surgency from the 18-month to 24-month assessment ($B$ =0.6 *p-value* = 0.03). Feeding variables and child BMI-z scores were stable across time, with no evidence of a time-by-group interaction.

**Table 3:** Characteristics of participants in the mealtime for toddlers study by depressive symptom trajectory group

	Sample (*n* = 111)	No-Minimal and Decreasing (*n* = 53)	Mild-Moderate and Stable (*n* = 46)	Moderate-Severe and Stable (*n* = 12)
**Mother**				
Age	30.6 ± 4.8	31.0 ± 0.8	31.0 ± 0.7	27.7 + 1.6
Ethnicity				
Hispanic	47 (42)	23 (44)	18 (40)	6 (50)
Non-Hispanic	64 (58)	30 (56)	28 (60)	6 (50)
Race^1^				
American Indian or Alaska Native	5 (5)	2 (4)	1 (3)	2 (17)
Asian	2 (2)	0 (0)	2 (4)	0 (0)
Black	5 (5)	4 (8)	1 (2)	0 (0)
Native Hawaiian or Pacific Islander	1 (1)	0 (0)	1 (2)	0 (0)
White	59 (54)	25 (47)	27 (59)	7 (58)
Other/ More than 1 race	33 (30)	19 (35)	11 (24)	3 (25)
Highest Level of Education				
College Degree	45 (41)	20 (37)	21 (46)	4 (33)
Less than College Degree	66 (59)	33 (63)	25 (54)	8 (67)
Income Level				
< 150% federal poverty line	47 (42)	25 (48)	14 (30)	8 (66)
> 150% federal poverty line	64 (58)	28 (52)	32 (70)	4 (33)
Marital Status				
Married or living with partner	100 (90)	48 (91)	44 (96)	8 (66)
Not married or living with partner	11 (10)	5 (9)	2 (4)	4 (33)
Food Security				
18-month assessment				
Food Insecure	25 (23)	6 (12)	13 (28)	6 (50)
Food Secure	86 (77)	47 (88)	33 (72)	6 (50)
24-month assessment				
Food Insecure	24 (22)	5 (9)	13 (28)	5 (42)
Food Secure	87 (78)	48 (91)	33 (72)	7 (58)
Healthy Beginnings/ Comienza Sano Group				
Intervention	56 (53)	25 (47)	25 (55)	9 (75)
Standard Care	50 (47)	28 (53)	20 (45)	3 (25)
Site				
CA	60 (54)	26 (49)	27 (59)	7 (58)
RI	51 (46)	27 (51)	19 (41)	5 (42)
**Child**				
Sex				
Female	57 (51)	26 (50)	24 (52)	7 (48)
Male	54 (49)	27 (50)	22 (48)	5 (42)
BMI z-score				
18-month assessment	0.7 ± 0.1	0.7 + 0.2	0.7 + 0.2	0.7 ± 0.3
24-month assessment	0.8 + 0.1	0.7 ± 0.1	0.8 ± 0.1	0.8 ± 0.2
Effortful control				
18-months	5.2 ± 0.1	5.2 ± 0.1	5.3 ± 0.1	5.2 ± 0.2
24-months	5.7 ± 0.1	5.5 ± 0.1	5.6 ± 0.1	5.7 ± 0.2
Negative affect				
18-months	4.1 ± 0.1	4.0 ± 0.1	4.2 ± 0.2	4.3 ± 0.2
24-months	4.2 ± 0.1	4.2 ± 0.2	4.3 ± 0.2	4.0 ± 0.4
Surgency				
18-months	5.6 ± 0.1	5.5 ± 0.1	5.7.0.1	5.5 ± 0.2
24-months	5.7 + 0.1	5.5 ± 0.1	5.8 ± 0.1	6.1 ± 0.2

Mean ± Standard Error presented for continuous variables n (%) presented for categorical variables. ^1^ Participants self-identified race as American Indian or Alaska Native, Asian, Black, Native Hawaiian or Pacific Islander, White, or Other. 5 participants declined to respond to this item

**Table 4:** Self-reported and observed child feeding by depressive symptom trajectory group at 18 and 24-months postpartum

	No-Minimal and Decreasing (*n* = 53)	Mild-Moderate and Stable (*n* = 46)	Moderate-Severe and Stable (*n* = 12)
	Mean ± Std	Mean ± Std	Mean ± Std
Meal Observation			
Maternal Responsiveness to Child Satiation Cues			
18-months	3.1 ± 0.2	3.2 ± 0.2	4.2 ± 0.3
24-months	3.4 ± 0.2	3.3 ± 0.2	4.0 ± 0.3
Child Feeding Questionnaire			
Pressure to eat			
18-months	2.7 ± 0.1	2.6 ± 0.1	2.1 ± 0.3
24-month	2.7 ± 0.1	2.7-±0.1	2.5 ± 0.3
Restriction			
18-months	3.0 ± 0.1	3.3 ± 0.1	3.4 ± 0.3
24-month	3.2 ± 0.1	3.3 ± 0.1	3.2 ± 0.3
Concern for Child Weight Status			
18-months	1.4 ± 0.1	1.5 ± 0.1	1.7 ± 0.3
24-months	1.4 ± 0.1	1.7 ± 0.1	1.9 ± 0.4

At the 18-month assessment, when compared to the No-Minimal and Decreasing group, membership in the Moderate-Severe and Stable group was associated with higher maternal responsiveness to child satiation cues ($B$ =2.3, *95%CI =* 0.2, 4.4) and lower self-reported pressure to eat ($B$=-0.4, *95%CI*= -0.7, 0.0). When compared to the No-Minimal and Decreasing group, membership in the Mild-Moderate and Stable group was associated with higher self-reported restriction ($B$ =0.4, *95%CI* = 0.0,0.7). Depressive trajectory group membership was not associated with self-reported or observed feeding practices at the 24-month assessment.

**Table 5:** Associations between maternal depressive symptom trajectory group and maternal feeding practices

	No-Minimal and Decreasing (*n* = 53)	Mild-Moderate and Stable (*n* = 46)	Moderate-Severe and Stable (*n* = 12)
Meal Observation		$B$ (*95% CI)*	$B$ *(95% CI)*
Maternal Responsiveness to Child Satiation Cues			
18-months	Reference	0.4 (-0.6, 1.5)	**2.3 (0.2 4.4)**
24-months	Reference	-0.7 (-1.8, 0.4)	0.7 (-1.0, 2.3)
Child Feeding Questionnaire^1^		B (95% CI)	B (95% CI)
Pressure to eat			
18-months	Reference	-0.1 (-0.2, 0.1)	**-0.4 (-0.7, -0.0)**
24-month	Reference	0.1 (-0.3, 0.5)	-0.3 (-0.9, 0.3)
Restriction			
18-months	Reference	**0.4 (0.0, 0.7)**	0.2 (-0.4, 0.8)
24-month	Reference	0.0 (-0.4, 0.4)	-0.3 (-0.9, 0.4)

Estimate and 95% confidence intervals were calculated using generalized linear^1^ and multinomial^2^ regressions. Models adjusted for intervention group, site, maternal age, ethnicity, education, household income, food insecurity, child gender, BMI z-score, and mother’s concern for child’s weight status. Associations significant at *p* < 0.05 are indicated in bold font

### Associations between continuous mean BDI-II score and child temperament with feeding

Correlations between continuous variables are shown in Supplemental Table 1. Mean BDI-II scores were positively correlated with concern for child weight status and child surgency at 24 months (*r* = 0.34, *p-value* = 0.001 and *r* = 0.23, *p-value* = 0.02, respectively). Mean BDI-II scores were not significantly correlated with self-reported or observed feeding.

Effortful control at 18 months was negatively correlated with maternal responsiveness to satiation cues at 24 months (*r*=-0.24, *p-value* = 0.048). Surgency was positively correlated with restriction at 24-months (*r* = 0.21, *p-value* = 0.04).

There was an interaction between mean BDI-II score and child effortful control on the association with restriction at 18 months, $B=0.2, 95\% CI (0.0, 04)$; whereby greater maternal depressive symptoms predicted greater use of restriction when children had high effortful control (1 standard deviation greater than the mean), $B=0.1, p-value=0.01$(Supplemental Fig. 1).

There was an interaction between mean BDI-II score and child surgency in the association with restriction at 24 months $B=-0.3, 95\% CI (-0.6, 0.0)$; with greater maternal depressive symptoms associated with greater use of restriction when children had low surgency (1 SD less than the mean), $B=0.1, p=0.06$(Supplemental Fig. 2).

## Discussion

In this analysis, we found that mothers who participated in a prenatal lifestyle randomized controlled trial exhibited three distinct trajectories of depressive symptoms through the first two years postpartum. The three trajectories were characterized primarily by the severity of symptoms. While we found some evidence that a trajectory with elevated depressive symptoms were associated with greater restriction at 18 months, our results overall did not support the hypothesis that elevated depressive symptoms are associated with less responsive feeding as assessed via observation and self-report. We did however find that the association between maternal depressive symptoms and use of restriction may be moderated by their child’s temperament.

Our finding that mothers who participated in the HB/CS study exhibited three distinct depressive symptom trajectories characterized by severity is consistent with the broader literature on postpartum depressive symptomatology [51, 57, 58]. While many have hypothesized that some women may be more likely to experience an increase in depressive symptomology in the postpartum period, our data and that of others suggest that women who experience higher levels of depressive symptoms postpartum have likely had pre-existing elevated depressive symptoms [57–61]. A systematic review of longitudinal studies on depressive symptoms found that the majority of individuals experience minimal depressive symptoms, with a minority of ~ 10% experiencing elevated symptoms, associated with female gender, low income and education, and non-white race [60]. While most women in this study experienced minimal depressive symptoms during pregnancy which significantly decreased over the postpartum period, women who had mild and moderate symptoms saw them persist. This highlights the need for depressive screening prenatally to identify mothers with elevated depressive symptoms, rather than waiting until the post-partum period. It may also be warranted to monitor women with mild depressive symptoms which, if persistent, may impact the mother/child feeding relationship.

Membership in the Moderate-Severe and Stable trajectory group was not associated with lower responsiveness to satiation cues, greater pressure to eat, or greater restriction. Surprisingly, we found that mothers in the Moderate-Severe and Stable group were more responsive to their child’s satiation cues at the 18-month assessment, as assessed by video observation and self-report. However, it is important to acknowledge that this study was conducted in a sample of women who participated in a prenatal lifestyle intervention, and few (*n* = 12) experienced Moderate-Severe and Stable trajectories. Thus, these results are not representative of women with elevated depressive symptoms broadly and, given the small number in this trajectory group, needs to be interpreted with some caution. A meta-analysis exploring the association between depressive symptoms and maternal sensitivity to infant cues found a small but meaningful association (*r*=-0.16, *p* < 0.0001), and demonstrated that the effect was larger when studies examined differences between mothers with and without clinical depression (-0.35, *p* < 0.0001) [62]. Additionally, satiation cues generally become easier to recognize from infancy throughout toddlerhood, although satiation cues remain more challenging to identify as compared to hunger cues [28]. Impaired ability to recognize and respond to child satiation cues may be more pronounced in women with clinical depression or severe depressive symptoms (BDI-II score > 30), who were not represented in this sample [40].

Previous studies have found depressive symptoms to be associated with less responsiveness to child satiation cues, although the majority report assessing feeding at or over 24 months of age [14, 27, 31]. For example, Mallan et al. found that depressive symptoms in the postpartum period (4 months) were associated with greater self-reported pressure to eat at 24 months [31], and Elias et al. found a cross-sectional association between depressive symptoms and observed pressure to eat [63]; both adjusting for caregiver perception of difficult child temperament. While maternal pressure to eat is generally reported to be stable over time [64], as seen in our data, mothers in the moderate-severe and stable? depressive symptom group had higher observed responsiveness to satiation cues and lower self-reported pressure to eat at the 18-month, but not 24-month assessment suggesting that there may be a change in the association between depressive symptoms on feeding that develops as children grow. Blisset et al. also found different associations across time points, with results demonstrating that depressive symptoms at 12 months predicted higher use of pressure at 24 months, but not at 12 months [53]. Taken together these results provide evidence that women who have stable trajectories of mild or moderate depressive symptoms can be responsive to their child’s satiation cues, however, the impact of their depressive symptoms on their feeding practices may change as children grow.

One potential explanation for this potential change is that as children approach 24 months of age, their growth rate begins to slow, resulting in decreased or more erratic appetite compared to infancy. Concurrently, children are developing effortful control (goal-oriented, volitional behavior), which typically emerges towards the end of the first years of life [65, 66]. This was seen in our data as children’s effortful control, on average, increased from 18 to 24 months. The combination of decreased appetite and developing autonomy often results in children exerting more control regarding food choices and intake [67]. While these changes are developmentally appropriate, many mothers experience low parental self-efficacy and feelings of concern, frustration, and guilt when children refuse food at meals [68]. These feelings may be particularly salient for women who have already been experiencing chronic depressive symptoms, causing mothers to turn to less supportive feeding strategies [9].

Consistent with previous research on maternal depression and feeding practices, we did find evidence that, when compared to those in the No-Minimal and Decreasing group, those in the Mild-Moderate and Stable group reported greater use of restriction at 18 months [53]. However, this association was not significant for the Moderate-Severe and Stable group at 18 months nor for either group at the 24-month assessment. This finding may suggest that chronic, mild-moderate, depressive symptoms influence mothers’ use of restriction early in toddlerhood, which could increase the emotional valiance and intake of foods that are restricted and lead to poorer long-term self-regulation for some children [54].

It is important to note however that the utility of restrictive feeding practices can depend on contextual factors such as the way that they are implemented and the individual temperament of the child. Overt restriction without regard for the feelings of the child, such as withholding palatable foods others are consuming, can promote future intake of that food [69]. However, more covert strategies assessed in the CFQ such as modifying the visibility and accessibility of foods (“I intentionally keep some foods out of my child’s reach”) and awareness of the impact of foods that are high in sugar and added fats (“I have to be sure that my child does not eat too many sweets (candy, ice cream, cake, pastries)”) can be achieved by controlling the feeding environment without controlling the child’s behavior. In fact, these strategies may be protective for children who have less inhibitory control and/or are more responsive to their environment [70]. There is an emerging body of research that suggests that the feeding relationship between caregiver and child is bi-directional, with children shaping their parents’ use of feeding practices [71–73]. Indeed, an important characteristic of responsive parenting is that the caregiver’s responses should be contingent on their child’s cues and behavior [74–76]. For example, caregivers of children who are very responsive to their environment may respond to constant requests for sweets by using restriction around the foods they bring into the home to prevent excess weight gain [76, 77]. We did see evidence of this in our data, with mother’s concern for their child’s weight positively correlated with restriction at both time points. We also saw that child surgency was positively correlated with restriction at 24-months, perhaps in response to their child’s behavior, as surgency is associated with increased eating in response to external cues, higher desire to eat, and more pleasure derived from food [78, 79].

Interestingly, in our exploratory analysis, continuous mean depressive symptom scores were positively associated with restriction when children had high levels of effortful control (18 months) and low levels of surgency or responsiveness to their environment (24 months). This could reflect a mismatch between feeding strategies and the needs of the child; when depressive symptoms are higher, mothers reported greater restriction if their children had temperaments not associated with increased obesogenic eating behaviors [80]. The impact of this mismatch may be compounded by the fact that mothers in the Moderate group reported an increase in child surgency from 18 to 24 months. This is a trend seen in other studies of mothers with depression such that children with higher surgency may benefit from more maternal control of the feeding environment. This analysis looked at mean continuous BDI scores, as we were not powered to evaluate the complex bi-directional, direct, and indirect relationships between maternal depressive symptom trajectory, child temperament, and responsive feeding. However, these exploratory results suggest that depressive symptoms may impact mothers’ ability to adjust their feeding strategies to the temperament of their child.

The limitations of the study are important to note. First, MTT is a sample of women who participated in the HB/CS randomized trial, therefore all women had obesity and consented to be randomized to a multi-component lifestyle intervention or usual prenatal care during pregnancy. While understanding the role of depressive symptoms and child feeding is important in this high-risk population, these results cannot be generalized to mothers without obesity or mothers with obesity who were not interested in participating in a prenatal lifestyle intervention. Mothers who were interested in making lifestyle changes to promote weight loss may have lived experiences and beliefs about weight, eating, and health that could influence how depressive symptoms relate to their food parenting behaviors. While we were able to objectively assess maternal responsiveness to satiation cues using video observation, ICC was acceptable but indicative of some disagreement between coders [81]. Additionally, the sample size was relatively small, and depressive symptoms in the sample were minimal on average. While we were able to examine the impact of child temperament on the relationship between depressive symptoms and feeding, these analyses were exploratory and meant to be hypothesis-generating. As we were not testing a-priori hypotheses we acknowledge that significant associations may be false positives and that more research, with larger samples that target women with clinical depression, may be needed to uncover the true nature of the relationship between maternal depressive symptoms, child temperament, and feeding. In light of these limitations, this study has several strengths including rich, longitudinal data on a diverse sample of women whose children are at increased risk for developing obesity. The collection of both self-reported and observed maternal feeding, and consistency between the associations with depressive symptom trajectory strengthens our ability to interpret these findings.

This study provides valuable insight into the association between maternal depression and child feeding and temperament. We found that, contrary to our hypothesis, a moderate-severe and stable? maternal depressive symptom trajectory was not associated with less responsive feeding practices. We did find evidence that the Mild-Moderate and Stable group was associated with greater self-reported restrictive feeding practices at 18 months and preliminary evidence that maternal depressive symptoms may impair mothers’ ability to match their feeding strategies to their child’s unique temperament. It is reassuring that stable mild-moderate and moderate-severe depressive symptoms did not impair women in this study’s ability to recognize and respond to their young child’s satiation cues. However, future efforts to understand and promote mutually responsive feeding between mothers with obesity and their children should consider the impact of depressive symptoms on mother’s use of restriction, and how it relates to the unique temperament of their child.

### Electronic supplementary material

Below is the link to the electronic supplementary material.

Supplementary Material 1

## Data Availability

The data that support the findings of this study are not openly available due to reasons of sensitivity and are available from the corresponding author upon reasonable request.

## Abbreviations

HB/CS: Healthy Beginnings/ Comienzos Saludables
MTT: Mealtime for Toddlers
BDI-II: Beck Depression Inventory II
RCFCS: Responsiveness to Child Feeding Cue Scale
ICCs: Intraclass correlation coefficients
CFQ: Child Feeding Questionnaire
IBQR VSF: Infant Behavior Questionnaire-Revised Very Short Form
GBTM: Group-based trajectory modeling
BIC: Bayesian Information Criterion
APPA: Average Posterior Probability of Assignment
