# Supplementary material for: Maternal depressive symptom trajectories and associations with child feeding
Source: BMC Public Health. 2024 Jun 19;24:1636. doi: 10.1186/s12889-024-19110-8 (PMC11186209; doi:10.1186/s12889-024-19110-8)
Supplement: Supplementary file 1 — Supplementary Material 1 [file 12889_2024_19110_MOESM1_ESM.docx]

| Supplemental Table 1 Correlations Between Analytic Variables | | | | | | | | | | | | | | | | |
| --- | --- | --- | --- | --- | --- | --- | --- | --- | --- | --- | --- | --- | --- | --- | --- | --- |
|  | 1 | 2 | 3 | 4 | 5 | 6 | 7 | 8 | 9 | 10 | 11 | 12 | 13 | 14 | 15 | 16 |
| 1. Mean BDI | 1 |  |  |  |  |  |  |  |  |  |  |  |  |  |  |  |
| 2.Responsiveness (18-months) | 0.17 (0.10) | 1 |  |  |  |  |  |  |  |  |  |  |  |  |  |  |
| 3.Responsiveness (24-months) | 0.09 (0.43) | 0.19 (0.23) | 1 |  |  |  |  |  |  |  |  |  |  |  |  |  |
| 4. Pressure to eat (18-months) | -0.14 (0.18) | -0.22 (0.06) | -0.13 (0.33) | 1 |  |  |  |  |  |  |  |  |  |  |  |  |
| 5 Pressure to eat (24-months) | -0.07 (0.47) | -0.04 (0.72) | -0.16 (0.18) | **0.42 (<0.001)** | 1 |  |  |  |  |  |  |  |  |  |  |  |
| 6. Restriction (18-months) | 0.18 (0.09) | -0.05 (0.66) | -0.10 (0.39) | **0.29 (0.0)** | 0.18 (0.13) | 1 |  |  |  |  |  |  |  |  |  |  |
| 7. Restriction (24-months) | 0.04 (0.68) | 0.00 (0.99) | -0.08 (0.52) | 0.23 (0.07) | **0.34 (0.002)** | **0.37 (0.001)** | 1 |  |  |  |  |  |  |  |  |  |
| 8. Concern (18-months) | 0.20 (0.06) | -0.10 (0.39) | -0.19 (0.14) | -0.04 (0.72) | -0.08 (0.49) | **0.22 (0.04)** | **0.25 (0.02)** | 1 |  |  |  |  |  |  |  |  |
| 9. Concern (24-months) | **0.34 (0.001)** | 0.02 (0.87) | -0.09 (0.48) | -0.09 (0.41) | -0.07 (0.52) | 0.20 (0.07) | **0.27 (0.008)** | **0.87 (<0.001)** | 1 |  |  |  |  |  |  |  |
| 10. Surgency (18-months) | -0.04 (0.68) | -0.13 (0.25) | 0.00 (0.99) | 0.06 (0.58) | -0.04 (0.73) | 0.07 (0.50 | 0.11 (0.37) | 0.09 (0.40) | 0.11 (0.29) | 1 |  |  |  |  |  |  |
| 11. Surgency (24-months) | **0.23 (0.02)** | 0.01 (0.92) | 0.01 (0.92) | -0.19 (0.08) | 0.08 (0.43) | -0.12 (0.24) | **0.21 (0.04)** | 0.16 (0.13) | 0.15 (0.15) | **0.53 (<0.001)** | 1 |  |  |  |  |  |
| 12. Negative Affectivity (18-months) | 0.02 (0.39) | -0.11 (0.34) | 0.07 (0.54) | 0.02 (0.88) | 0.03 (0.79) | 0.03 (0.76) | 0.16 (0.16) | 0.06 (0.54) | 0.15 (0.17) | **0.26 (0.03)** | **0.30 (0.005)** | 1 |  |  |  |  |
| 13. Negative Affectivity (24-months) | 0.02 (0.83) | -0.04 (0.71) | 0.00 (0.99) | -0.12 (0.27) | 0.11 (0.30) | 0.04 (0.75) | 0.17 (0.12) | 0.02 (0.83) | 0.13 (0.22) | **0.23 (0.04)** | **0.36 (<0.001)** | **0.52 (<0.001)** | 1 |  |  |  |
| 14. Effortful Control (18-months) | -0.03 (0.76) | -0.12 (0.31) | **-0.24 (0.048)** | -0.09 (0.39) | -0.05 (0.66) | 0.05 (0.64) | 0.09 (0.46) | 0.10 (0.34) | 0.05 (0.64) | **0.56 (<0.001)** | **0.33 (0.002)** | -0.04 (0.66) | 0.04 (0.70) | 1 |  |  |
| 15. Effortful Control (24-months) | 0.09 (0.38) | -0.13 (0.27) | 0.02 (0.90) | -0.12 (0.27) | 0.06 (0.57) | -0.03 (0.80) | 0.18 (0.08) | 0.13 (0.25) | 0.11 (0.31) | **0.21 (0.07)** | **0.55 (<0.001)** | 0.04 (0.72) | 0.14 (0.18) | **0.54 (<0.001)** | 1 |  |
| 16. BMI-z Score 18-months | 0.02 (0.87) | 0.02 (0.85) | -0.06 (0.61) | 0.07 (0.51) | -0.09 (0.41) | 0.17 (0.11) | 0.20 (0.08) | **0.26 (0.01)** | **0.25 (0.01)** | 0.14 (0.17) | **0.23 (0.03)** | 0.04 (0.70) | 0.12 (0.24) | 0.14 (0.18) | 0.14 (0.18) | 1 |
| 17. BMI-z Score 24-months | -0.01 (0.92) | 0.08 (0.49) | 0.01 (0.91) | 0.11 (0.34) | -0.07 (0.51) | 0.14 (0.22) | **0.23 (0.03)** | 0.21 (0.07) | 0.12 (0.24) | 0.06 (0.57) | 0.17 (0.08) | 0.06 (0.60) | 0.12 (0.25) | -0.03 (0.80) | 0.01 (0.89) | **0.87 (0.001)** |
|  | | | | | | | | | | | | | | | | |

| Supplemental Table 2: Interaction Effects Between Depressive Symptoms and Child Temperament on the Association with Maternal Feeding Practices | | | | |
| --- | --- | --- | --- | --- |
|  | Mean BDI | Mean BDI x surgency | Mean BDI x negative affectivity | Mean BDI x effortful control |
|  | B (95%CI) | B (95%CI) | B (95%CI) | B (95%CI) |
| Maternal Responsiveness to Child Satiation Cues |  |  |  |  |
| 18-months | 0.5 (0.0, 1.0) | 0.1(-0.7, 0.8) | 0.1 (-0.5, 0.7) | 0.0 (-0.6, 0.6) |
| 24-months | 0.1 (-0.4, 0.6) | 0.3 (-0.3, 1.0) | 0.0 (-0.4, 0.4) | 0.1 (-0.6, 0.7) |
| Child Feeding Questionnaire^1^ |  |  |  |  |
| Pressure to eat |  |  |  |  |
| 18-months | -0.2 (-0.4, 0.0) | 0.0 (-0.1, 0.1) | 0.0 (-0.1, 0.1) | 0.0 (-0.1, 0.1) |
| 24-month | 0.0 (-0.2, 0.2) | -0.2 (-0.5, 0.1) | 0.0 (-0.2, 0.2) | -0.2(-0.4, 0.1) |
| Restriction |  |  |  |  |
| 18-months | 0.0 (-0.0, 0.1) | 0.1 (-0.1, 0.4) | 0.0 (-0.3, 0.2) | **0.2(0.0-0.4)** |
| 24-month | 0.0 (-0.1, 0.0) | **-0.3 (-0.6, 0.0)** | 0.0 (-0.1, 0.2) | -0.1 (-0.3, 0.1) |
|  |  |  |  |  |
| Standardized B and 95% confidence intervals calculated using generalized linear^1^ and multinomial^2^, regressions. Bolded font indicating significant interaction at p<0.1. Models adjusted for intervention group, site, maternal age, ethnicity, education, household income, food insecurity, child gender, BMI z-score, and concern for child weight. | | | | |

Figure 1 Simple slope plot at two levels of child effortful control.

Beck Depression Inventory (BDI). Child Feeding Questionnaire (CFQ).

Significant slopes are represented by a solid line and non-significant slopes are represented by a dotted line.

Figure 2 Simple slope plot at two levels of child surgency.

Beck Depression Inventory (BDI). Child Feeding Questionnaire (CFQ).

Significant slopes are represented by a solid line and non-significant slopes are represented by a dotted line.
